# Real-world assessment of thromboembolic risk associated with tamoxifen

**DOI:** 10.1038/s41598-025-13585-0

**Published:** 2025-07-30

**Authors:** Jiajia Jia, Chunyu Tian, Wenchao Han, Qi Ma

**Affiliations:** 1 Department of Pharmacy, The Seventh People’s Hospital of Zhengzhou, Zhengzhou, 450000 Henan China; 2 Department of Pharmacy, The Sixth People’s Hospital of Zhengzhou, Zhengzhou, 450000 Henan China

**Keywords:** Tamoxifen, Thromboembolic events, FAERS, Disproportionality analyses, Adverse events, Drug regulation, Drug safety, Breast cancer, Data acquisition, Data mining, Data processing, Data publication and archiving, Databases, Literature mining

## Abstract

It has been confirmed that tamoxifen (TAM) use is associated with thromboembolic risks; however, it remains uncertain which specific thromboembolic events warrant prioritized surveillance beyond those already listed in the prescribing information. This study was conducted to assess the relationship between TAM use and reported thromboembolic events using the latest FDA Adverse Event Reporting System (FAERS) data. Tamoxifen-related AEs reported in FAERS between January 2004 and December 2024 were extracted, screened for thromboembolic cases, and subjected to disproportionality analysis to identify significant signals. A clinical prioritization scoring system was then applied to rank signal relevance, and multivariate regression analyses were performed to identify factors associated with thromboembolic outcomes. Among 385 TAM-associated thromboembolic events, 28 significant signals were detected. Newly detected signals comprised unilateral paralysis, retinal artery occlusion, and atrial thrombosis. Male sex, older age, and elevated body mass were identified as risk indicators for thromboembolism. These findings may inform enhanced clinical surveillance and TAM risk stratification; however, further validation is warranted.

**Supplementary Information:**

The online version contains supplementary material available at 10.1038/s41598-025-13585-0.

## Introduction

Since the 1970s, tamoxifen, a selective estrogen receptor modulator (SERM), has been employed as a cornerstone of adjuvant therapy for hormone receptor–positive breast cancer. It has been demonstrated by large-scale meta-analyses with 15-year follow-up that five-year tamoxifen therapy reduces recurrence risk by 39% and breast cancer mortality by 30%, and that extending therapy to ten years further enhances survival outcomes^1,2^. The mechanism of action is characterized by estrogen antagonism in breast tissue, while partial estrogen agonist activity is concurrently observed in other systems (e.g., cardiovascular and skeletal)^3^. This tissue-selective profile may be intimately linked to the observed thrombotic risk^4^.

According to clinical data, using tamoxifen raises the risk of venous thromboembolism (VTE) by two to three times^5^and the risk increases with treatment duration^4,6,7^. According to the NSABP P-1 trial, patients on tamoxifen had a higher risk of stroke, pulmonary embolism, and deep vein thrombosis, and these events were more prevalent in women 50 years of age or older^8^. Despite a clear clinical consensus regarding tamoxifen-associated thrombotic risk in female patients, the risk in male breast cancer (MBC) patients—who represent approximately 1% of all cases—has remained understudied^9^. It should be noted that current MBC treatment guidelines are derived from limited retrospective studies and extrapolated from female breast cancer (FBC) management, and that most ongoing breast cancer clinical trials continue to exclude male participants^10,11^. Given inherent physiological differences between sexes, direct application of FBC management strategies to MBC warrants caution. Furthermore, although thromboembolic events (TEs) are designated as a black box warning on tamoxifen labeling^12^the specific thrombus types and their clinical monitoring priorities remain undefined.

The FDA Adverse Event Reporting System (FAERS) is recognized as a global pharmacovigilance database^13^. In this study, a comprehensive pharmacovigilance analysis was conducted using FAERS data to investigate TAM-associated thromboembolic risk, encompassing sex-related differences and rare subtypes, with the aim of enhancing patient safety in real-world clinical practice.

## Materials and methods

### Data sources and processing

To ensure complete case inclusion, all seven structured FAERS data files were downloaded, including patient demographics (DEMO), drug exposure (DRUG), adverse events (REAC), outcomes (OUTC), report sources (RPSR), therapy dates (THER), and medical indications (INDI). All tamoxifen-associated reports from the first quarter of 2004 (Q1 2004) to the fourth quarter of 2024 (Q4 2024) were retrieved from the FAERS database through the official portal (https://fis.fda.gov/extensions/FPD-QDE-FAERS/FPD-QDE-FAERS.html).

In accordance with FDA guidelines for pharmacovigilance studies, a hierarchical deduplication strategy was implemented. Primary case identification numbers were used as the primary identifier, with duplicate entries being removed based on the highest report number and the most recent FDA receipt date. A search was conducted to identify cases related to TAM, with the following keywords employed as search criteria: including “TAMOXIFEN”, “SOLTAMOX”, and “NOLVADEX”. For the purpose of subsequent analysis, reports designating tamoxifen as the “Primary Suspect (PS)” were retained.

### Data mining

**Fig. 1:**
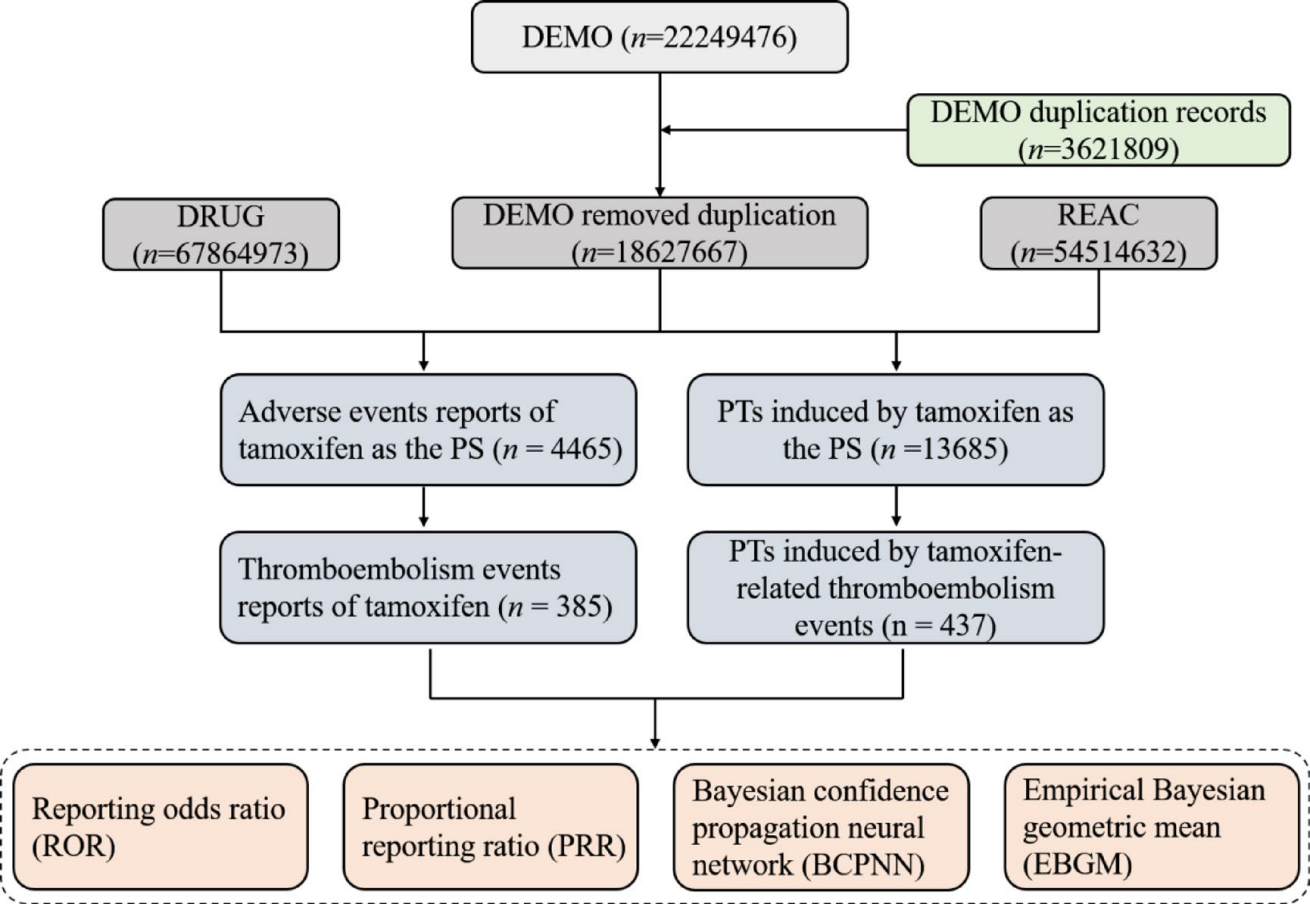
Flow diagram of the selection of TAM-related AEs from the FAERS database.

Baseline characteristics—including sex distribution, age categories, reporting region, and reporter type—were described using descriptive statistics. Thromboembolic events were identified via a Standardized MedDRA Query (narrow scope: Embolic and Thrombotic Events [SMQ 20000081]) and mapped to Preferred Terms (PTs; e.g., pulmonary embolism, deep vein thrombosis) and System Organ Classes (SOCs; e.g., eye disorders, blood and lymphatic system disorders). Non–drug-related PTs (e.g., malignancy-associated thrombosis, product quality issues) and reports with date inconsistencies (e.g., event dates preceding treatment initiation) were excluded. A 2 × 2 contingency table (Supplementary Table S1) was employed for disproportionality analysis, and signals were detected using four algorithms: Reporting Odds Ratio (ROR), Proportional Reporting Ratio (PRR), Bayesian Confidence Propagation Neural Network (BCPNN), and Empirical Bayes Geometric Mean (EBGM). Positive signals were defined as those meeting predefined significance thresholds across all four algorithms simultaneously (see Supplementary Table S2 for criteria). All data processing was performed in R (v4.3.1), with duplicate reports removed prior to statistical analysis, and the study flow diagram is presented in Fig. 1.

### Clinical prioritization of disproportionality signals

The clinical priority of TEs was assessed using a semi-quantitative scoring scale based on the European Medicines Agency lists of Designated Medical Events (DME) and Important Medical Events (IME)^14^. The system was stratified and evaluated on the following five dimensions: (1) Number of target events, (2) Lower limit of ROR, (3) Mortality proportion, (4) IMEs or DMEs and (5) Biological plausibility. Among them, IME is defined as a serious AE with standardized diagnostic criteria, and DME specifically refers to rare drug-related serious AEs that require immediate initiation of regulatory review. The scoring criteria for each dimension are detailed in Supplementary Table S3. Each dimension was categorized into three levels (0–2 points) according to preset thresholds, and only the highest score was taken when multiple criteria were met within the same dimension. A total score of 0–4 points is categorized as weak priority, 5–7 points as moderate priority, and 8–10 points as strong priority.

### Subgroup analysis and risk factor assessment

A subgroup analysis stratified by gender was conducted to systematically compare the distribution of tamoxifen-related thromboembolic events between sexes. Multivariable logistic regression was then performed to evaluate associations between selected risk factors (age, sex, and body weight) and the risk of thromboembolism, yielding adjusted odds ratios (ORs) with 95% confidence intervals. Due to substantial missingness and inconsistent coding in the FAERS database, important covariates such as comorbidities and concomitant medications could not be reliably included in the model. Therefore, only variables with high reporting completeness—namely, age, sex, and body weight—were retained to ensure analytical robustness. Two-sided tests were used for all analyses. To control for type I error due to multiple comparisons across 28 Preferred Terms, a Bonferroni-adjusted significance threshold of *p* < 0.001 was applied, despite the exploratory nature of signal detection.

## Results

### Descriptive analysis

This pharmacovigilance study utilized data from the US FDA Adverse Event Reporting System (FAERS) database (2004–2024). The raw dataset initially contained 22,249,476 demographic records (DEMO), from which 3,621,809 duplicate entries were removed using FDA-recommended deduplication protocols, resulting in 18,627,667 unique cases. Through linkage analysis with DRUG (*n* = 67,864,973) and REAC (*n* = 54,514,632), 4,465 adverse event (AE) reports were identified in which tamoxifen (TAM) was designated as the PS medication. Among these, 385 cases were specifically attributed to thromboembolic events directly associated with TAM therapy.

The characteristics of patients who suffered from TAM-related AEs are summarized in Table 1. A female predominance (78.7%) was observed among the 385 thromboembolic AE cases, slightly lower than the 84.0% female representation in all TAM-associated AEs (*n* = 4,465). The 50–64.9 age cohort accounted for 50.4% of thromboembolic events, exceeding the 44.9% prevalence in general TAM-related AEs. Notably, 31.7% of thromboembolism patients had body weights between 50 and 100 kg, compared to 21.9% in the broader AE cohort. Geographically, the United States contributed 37.7% of thromboembolism reports, with France (19.0%) and the UK (15.3%) comprising secondary clusters. Hospitalization rates for thromboembolic events (40.8%) substantially exceeded those for all AEs (17.6%). The mortality rate in the TE group (7%) was significantly higher than the overall rate (4.5%).

**Table 1:** Clinical characteristics of patients with TAM -associated AEs.

Characteristics number (%)	Thromboembolism AEs reported in TAM users (*n =* 385)	No-thromboembolism AEs reported in TAM users (*n =* 4080)	All AEs reported in TAM users (*n* = 4465)
Gender			
Female	303 (78.7%)	3448 (84.5%)	3751 (84.0%)
Male	43 (11.2%)	153 (3.8%)	196 (4.4%)
Missing data	39 (10.1%)	479 (11.7%)	518 (11.6%)
Age (years)			
< 45	23 (6.0%)	462 (11.4%)	485 (10.9%)
45 ≤ and ≤ 64.9	171 (44.4%)	1367 (33.5%)	1538 (34.4%)
65 ≤ and ≤ 85	99 (25.7%)	731 (17.9%)	830 (18.6%)
> 85	10 (2.6%)	39 (1.0%)	49 (1.1%)
Missing data	82 (21.3%)	1481 (36.3%)	1563 (35.0%)
Weight (kg)			
< 60	40 (10.39%)	399 (9.78%)	439 (9.83%)
60 ≤ and ≤ 80	58 (15.06%)	515 (12.62%)	573 (12.83%)
> 80	60 (15.58%)	208 (5.10%)	268 (6.00%)
Missing data	227 (58.96%)	2958 (72.50%)	3185 (71.33%)
Reporters			
Physician (MD)	142 (36.9%)	1228 (30.1%)	1370 (30.7%)
Pharmacist (PH)	50 (13.0%)	295 (7.2%)	345 (7.7%)
Health professional (HP)	21 (5.5%)	306 (7.5%)	327 (7.3%)
Consumer (CN)	72 (18.7%)	1405 (34.4%)	1477 (33.1%)
Lawyer (LW)	5 (1.3%)	17 (0.4%)	22 (0.5%)
Missing data	95 (24.7%)	829(20.3%)	924 (20.7%)
Reporter country (top three)			
America (US)	145 (37.7%)	1485 (36.4%)	1630 (36.5%)
United Kingdom (UK)	59 (15.3%)	625 (15.3%)	684 (15.3%)
France (FR)	73 (19.0%)	471 (11.5%)	544 (12.2%)
Time-to-onset (days)			
Median (IQR)	532 (1021)	470 (1015)	485 (1023)
Min-max	0-4960	0-8095	0-8095
Outcomes			
Death (DE)	27 (7.0%)	173 (4.2%)	200 (4.5%)
Disability (DS)	9 (2.3%)	200 (4.9%)	209 (4.7%)
Hospitalization-initial or prolonged (HO)	157 (40.8%)	629 (15.4%)	786 (17.6%)
Life-threatening (LT)	62 (16.1%)	118 (2.9%)	180 (4.0%)
Other serious outcome (OT)	105 (27.3%)	2077 (50.9%)	2182 (48.9%)
Missing data	25 (6.5%)	848 (20.8%)	908 (20.3%)

**Fig. 2:**
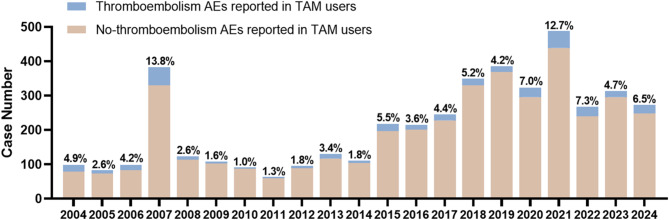
Yearly distribution of tamoxifen-associated thromboembolic events reported in the FAERS database (2004–2024).

After the FDA updated the black box warning for thrombotic risk in the drug labeling of TAM in 2005^12^, the number of TAM-related thromboembolic adverse reactions reported peaked in 2007 (Fig. 2), accounting for 13.8% of all adverse reactions reported in that year. Although the total number of TAM adverse reaction reports has been increasing year after year since then, the proportion of thromboembolic events has stabilized at about 5% after a period of increase. These data suggest that pharmacovigilance monitoring of TAM-induced TE should be strengthened in clinical practice.

### Disproportionality analysis

According to the SMQ narrow classification system, TAM-related thromboembolic events were classified into three categories: Arterial Thromboembolism (SMQ 20000082), Venous Thromboembolism (SMQ 20000084), and Unspecified Thromboembolism (SMQ 20000083). The study incorporated a total of 437 adverse event (AE) reports, of which 28 exhibited positive signals (meeting the four disproportionality analyses). The specific distributional characteristics are detailed in Table 2.

**Table 2:** Signal strength of PTs of TAM-related thromboembolism in FAERS database.

PT	*n*	ROR (95% Cl)	PRR (χ^2^)	IC (IC025)	EBGM (EBGM05)
Arterial thromboembolism (SMQ)					
Ischaemic stroke	20	4.88 (3.15–7.57)	4.88 (61.58)	2.28 (1.65)	4.87 (3.37)
Peripheral artery thrombosis	12	36.87 (20.88–65.11)	36.84 (414.58)	5.19 (4.39)	36.51 (22.69)
Blindness transient	7	4.20 (2.00–8.81)	4.19 (17.01)	2.07 (1.05)	4.19 (2.25)
Retinal artery occlusion	6	10.80 (4.85–24.07)	10.79 (53.18)	3.43 (2.34)	10.77 (5.51)
Coronary artery embolism	3	44.05 (14.12–137.45)	44.04 (124.8)	5.45 (3.99)	43.57 (16.81)
Venous thromboembolism (SMQ)					
Pulmonary embolism	123	5.59 (4.68–6.67)	5.54 (458.26)	2.47 (2.21)	5.54 (4.77)
Deep vein thrombosis	79	5.17 (4.14–6.45)	5.14 (263.54)	2.36 (2.04)	5.14 (4.27)
Cerebral venous thrombosis	24	58.10 (38.82–86.97)	58.00 (1325.13)	5.84 (5.26)	57.18 (40.8)
Pulmonary thrombosis	10	4.06 (2.18–7.54)	4.05 (22.98)	2.02 (1.15)	4.05 (2.41)
Portal vein thrombosis	9	12.54 (6.52–24.13)	12.53 (95.19)	3.64 (2.73)	12.49 (7.22)
Superior sagittal sinus thrombosis	7	36.91 (17.53–77.71)	36.89 (242.20)	5.19 (4.17)	36.56 (19.61)
Embolism venous	6	8.89 (3.99–19.82)	8.89 (41.92)	3.15 (2.06)	8.87 (4.54)
Thrombophlebitis	6	7.23 (3.24–16.11)	7.23 (32.13)	2.85 (1.76)	7.21 (3.69)
Jugular vein thrombosis	6	14.98 (6.72–33.40)	14.98 (77.96)	3.90 (2.81)	14.92 (7.63)
Budd-Chiari syndrome	5	88.50 (36.47–214.75)	88.47 (422.98)	6.44 (5.24)	86.56 (41.23)
Retinal vein occlusion	5	7.93 (3.30–19.07)	7.93 (30.21)	2.98 (1.80)	7.91 (3.80)
Venous thrombosis limb	5	9.62 (4.00–23.15)	9.62 (38.53)	3.26 (2.08)	9.60 (4.61)
Mesenteric vein thrombosis	4	17.64 (6.61–47.11)	17.64 (62.49)	4.13 (2.84)	17.56 (7.72)
Subclavian vein thrombosis	4	14.93 (5.59–39.87)	14.93 (51.79)	3.89 (2.60)	14.88 (6.54)
Transverse sinus thrombosis	4	32.23 (12.05–86.24)	32.22 (120.04)	5.00 (3.70)	31.97 (14.03)
Superficial vein thrombosis	4	29.81 (11.15–79.73)	29.80 (110.51)	4.89 (3.59)	29.59 (12.99)
Ophthalmic vein thrombosis	3	55.84 (17.86–174.53)	55.82 (159.28)	5.78 (4.33)	55.06 (21.22)
Vena cava thrombosis	3	7.78 (2.51–24.16)	7.78 (17.7)	2.96 (1.51)	7.77 (3.01)
Axillary vein thrombosis	3	36.04 (11.56–112.33)	36.03 (101.26)	5.16 (3.71)	35.72 (13.8)
Unspecified thromboembolism (SMQ)					
Thrombosis	62	3.35 (2.61–4.30)	3.34 (101.6)	1.74 (1.37)	3.34 (2.71)
Monoplegia	8	7.94 (3.97–15.89)	7.93 (48.38)	2.99 (2.02)	7.92 (4.43)
Antiphospholipid syndrome	6	16.93 (7.59–37.76)	16.93 (89.53)	4.08 (2.98)	16.86 (8.62)
Atrial thrombosis	3	6.12 (1.97–18.99)	6.12 (12.82)	2.61 (1.17)	6.11 (2.37)

**Fig. 3:**
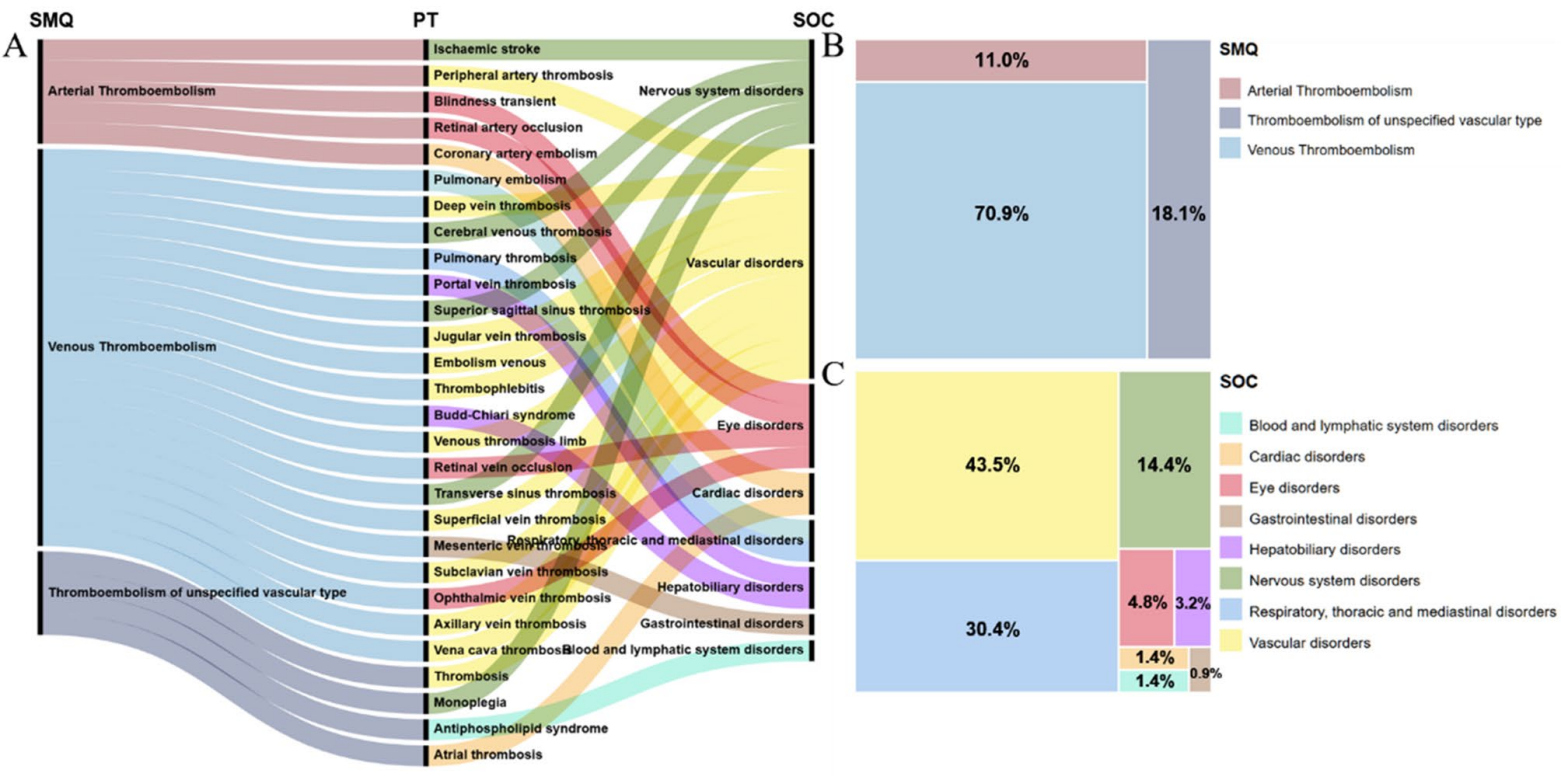
Disproportionality analysis of TAM. (**A**) Sankey diagram illustrating the hierarchical flow from Standardized MedDRA Queries (SMQs) to Preferred Terms (PTs) and subsequently to System Organ Classes (SOCs). Each band width reflects the number of cases assigned to a given category. (**B**) The proportion of TAM-related thromboembolism AE reports classified by SMQ. (**C**) The proportion of TAM-related thromboembolism AE reports classified by SOC.

The Sankey diagram (Fig. 3A) demonstrated a substantial discrepancy in the distribution of the 28 PTs between SMQ classification and SOC. The category of venous thromboembolism exhibited the highest proportion (70.9%, 310/437) (Supplementary Table S4), encompassing 19 PTs (Fig. 3B), with Pulmonary Embolism (*n* = 123, ROR = 5.59) and Deep Vein Thrombosis (*n* = 79, ROR = 5.17) as the predominant event types. Notably, Budd-Chiari syndrome (*n* = 5, ROR = 88.50), cerebral venous thrombosis (*n* = 24, ROR = 58.1), and ophthalmic vein thrombosis (*n* = 3, ROR = 55.84) showed higher signal intensity.

Arterial thromboembolic events accounted for 11.0% (48/437) of events involving 5 PTs, dominated by ischemic stroke (*n* = 20, ROR = 4.88) and peripheral artery thrombosis (*n* = 12, ROR = 36.87), with coronary artery embolism (*n* = 3, ROR = 44.05) presenting a significant risk signal. Unclassified thromboembolic events accounted for 18.1% (79/437) of cases and were dominated by thrombosis (*n* = 63, ROR = 3.35), with antiphospholipid syndrome (*n* = 6, ROR = 16.93) presenting a particularly prominent risk signal.

At the SOC level (Fig. 3C), the majority of PT subtypes were concentrated in the categories of Vascular disorders, Nervous system disorders, and Eye disorders. Conversely, respiratory, thoracic, and mediastinal disorders, comprising only two PTs, accounted for 30.4% (133/437) of cases. This is attributable to the high prevalence of pulmonary embolism in this cohort. Supplementary Table S5 presents a detailed exposition of the specific distribution characteristics of the SOC.

### Clinical prioritization of the disproportionality signals

As shown in Supplementary Table S6, among the 28 positive signals related to TEs, a total of 22 PTs (78.57%) was classified as IMEs. The priority stratification indicated that 16 AEs (57.1%) had a moderate clinical priority (scores 5–7), while 12 AEs (42.9%) had a weak priority (scores 0–4). Among the moderate-priority AEs, Pulmonary Embolism (*n* = 123, ROR_025_ = 4.68, score = 6), Deep Vein Thrombosis (*n* = 79, ROR_025_ = 4.14, score = 6), Thrombosis (*n* = 62, ROR_025_ = 2.61, score = 6), Coronary Artery Embolism (*n* = 3, ROR_025_ = 14.12, score = 6) and Cerebral Venous Thrombosis (*n* = 24, ROR_025_ = 38.82, score = 6) ranked in the top five. The AE type with the highest number of deaths was Pulmonary Embolism (*n* = 10), followed by Deep Venous Thrombosis (*n* = 8). Notably, the highest mortality rate (100%) was observed in Coronary Artery Embolism. New thromboembolic signals included Monoplegia (ROR_025_ = 3.97), Retinal Artery Occlusion (ROR_025_ = 4.85), and Atrial Thrombosis (ROR_025_ = 1.97). Their clinical significance was underscored by quantitative indicators (ROR values, number of deaths, scores) and structured classification.

### Subgroup analysis and risk factor assessment

Table 3 delineates the gender-stratified ROR (with 95% confidence intervals) for TEs, categorized into arterial (ATE), venous (VTE), and unspecified thromboembolism. Males exhibited significantly higher ROR than females in most TE subtypes. For example, in VTE subtypes, males demonstrated higher risks for pulmonary embolism (ROR = 15.98 vs. 5.09 in females) and deep vein thrombosis (ROR = 12.05 vs. 4.65). In particular, in ATE subtypes, coronary artery embolism showed a striking male predominance (ROR = 851.62). In addition, certain thromboembolic subtypes lacked sex-specific ROR estimates, particularly in males. This was not due to biological exclusivity, but rather to low reporting frequencies or zero-event counts in the FAERS database, which precluded stable estimation. These missing data points likely reflect underreporting or the rarity of these events, rather than the absence of risk.

**Table 3:** Gender-stratified TEs.

PT	Famale		Male	
	*n*	ROR (95% Cl)	*n*	ROR (95% Cl)
Arterial thromboembolism (ATE)				
Ischaemic stroke	17	6.07 (3.77–9.77)	3	13.61 (4.38–42.34)
Peripheral artery thrombosis	7	30.71 (14.57–64.71)		
Retinal artery occlusion	6	16.09 (7.21–35.9)		
Blindness transient	5	3.78 (1.57–9.08)		
Coronary artery embolism			3	851.62(269.8-2688.10)
Venous thromboembolism (VTE)				
Pulmonary embolism	96	5.09 (4.16–6.22)	15	15.98 (9.57–26.69)
Deep vein thrombosis	60	4.65 (3.61–6.00)	8	12.05 (6.00–24.22)
Cerebral venous thrombosis	19	48.48 (30.78–76.34)		
Pulmonary thrombosis	9	4.15 (2.16–7.98)		
Superior sagittal sinus thrombosis	6	31.47 (14.07–70.39)		
Thrombophlebitis	6	8.33 (3.74–18.56)		
Budd-Chiari syndrome	5	105.09 (42.98–256.96)		
Portal vein thrombosis	5	11.45 (4.76–27.57)	3	70.86 (22.76–220.63)
Retinal vein occlusion	5	10.44 (4.34–25.14)		
Venous thrombosis limb	5	11.50 (4.78–27.69)		
Embolism venous	4	9.12 (3.42–24.35)		
Transverse sinus thrombosis	4	26.08 (9.74–69.83)		
Superficial vein thrombosis	4	46.70 (17.37–125.55)		
Jugular vein thrombosis	3	7.79 (2.51–24.21)		
Unspecified thromboembolism				
Thrombosis	51	3.37 (2.56–4.43)	8	9.31 (4.63–18.71)
Monoplegia	6	6.86 (3.08–15.30)		
Antiphospholipid syndrome	3	8.84 (2.85–27.48)		

To gain a deeper insight into potential influencing factors, a multivariate regression analysis was conducted based on reports of thromboembolism associated with TAM. The significant predictors of risk of thromboembolic events are shown in Table 4. In a multivariate regression analysis, male sex, advanced age and high weight were significantly associated with the risk of thromboembolic events. Male gender was associated with a 2.97-fold increase in risk compared to female gender (*p* < 0.001). Each higher age category was associated with an exponential increase in risk, with the > 85 years age group showing an 11.92-fold increase in risk (*p* < 0.001). Weight > 80 kg was independently associated with increased risk of thromboembolic events (OR = 2.71, 95% CI: 1.70–4.30, *p* < 0.001), whereas the 60–80 kg group showed no significant association (OR = 1.03, 95% CI: 0.66–1.60, *p* = 0.901). The intercept term indicated low baseline risk in the reference group (OR = 0.02, 95% CI 0.01–0.06).

**Table 4:** The factors influencing TAM-related TEs.

Variables	Exp (beta)	S.E	Z	*P*	OR (95% CI)
Intercept	− 3.84	0.48	− 7.99	< 0.001	0.02 (0.01–0.06)
Gender					
Female (reference)					
Male	1.09	0.33	3.29	< 0.001	2.97 (1.55–5.68)
Age (years)					
< 45 (Reference)					
45 ≤ and ≤ 64.9	1.60	0.47	3.38	< 0.001	4.96 (1.96–12.55)
65 ≤ and ≤ 85	1.87	0.48	3.88	< 0.001	6.50 (2.53–16.71)
> 85	2.48	0.66	3.76	< 0.001	11.92 (3.28–43.35)
Weight (kg)					
< 60 (Reference)					
60 ≤ and ≤ 80	0.03	0.23	0.12	0.901	1.03 (0.66–1.60)
> 80	1.00	0.24	4.22	< 0.001	2.71 (1.70–4.30)

The considered exposure factors include gender, age, and weight.

*OR* odds ratio, *CI* confidence interval.

## Discussion

It has been demonstrated in early clinical studies^18^ that tamoxifen use is associated with increased mortality from stroke and pulmonary embolism. In the present study, demographics, temporal trends, and clinical heterogeneity of TAM-associated TE were systematically characterized by analyzing 385 thromboembolic events among 4,465 TAM-related adverse reports. Key findings included: (1) increased thrombotic susceptibility among men, older adults, and individuals with elevated body mass; (2) novel thromboembolic signals, including monoplegia, retinal artery occlusion, and atrial thrombosis; and (3) identification of high-risk thrombotic subtypes prioritized for clinical monitoring.

Women accounted for 78.7% of thromboembolic cases, a finding consistent with data from registry studies: the incidence of thromboembolism was higher in patients with FBC than in MBC^15^ after treatment with TAM, but the adjusted risk was elevated in men by a factor of 2.97, especially in arterial events (e.g., coronary artery embolism: male ROR = 851.62). This paradoxical phenomenon may be related to the lower basal estrogen levels in men and the exacerbation of the thrombogenic pathway by the partial estrogen agonism of tamoxifen^16^. The thrombogenic potential of tamoxifen is likely multifactorial. While it functions as an estrogen receptor antagonist in breast tissue, tamoxifen also exhibits partial estrogen agonist activity in hepatic and vascular tissues^17,18^. Studies have demonstrated that tamoxifen upregulates the synthesis of procoagulant factors (e.g., fibrinogen, prothrombin) and downregulates natural anticoagulants such as protein S^19,20^. In vascular endothelial cells, it has been shown to enhance the expression of adhesion molecules and promote platelet aggregation, further contributing to a prothrombotic state^21^. These effects may be particularly pronounced in male patients, whose low baseline estrogen levels could amplify the thrombogenic consequences of tamoxifen’s estrogenic activity.

The observed discrepancy between our findings and previous reports of TAM’s cardiovascular benefits may be explained by differences in the types of vascular outcomes assessed. Most earlier studies focused on chronic atherosclerotic endpoints (e.g., myocardial infarction)^22,23^whereas our analysis identified acute embolic events (e.g., coronary artery embolism, cerebral venous thrombosis). Furthermore, real-world users, including off-label male recipients, may differ substantially from postmenopausal women enrolled in clinical trials, further contributing to divergent risk profiles. These findings underscore the importance of stratified risk evaluation and warrant further mechanistic investigations. In addition MBC has a worse prognosis and lower overall survival compared to FBC, even after adjusting for age, race and treatment^24,25^suggesting the need for gender-specific risk management programs.

The age-related risk increased exponentially, with patients > 85 years of age showing an 11.92-fold increase in thromboembolic risk compared with those < 45 years (95% CI: 3.28–43.35). This trend is consistent with previous studies^26^ in which people aged 50-64.9 years accounted for 50.4% of thrombotic events. A threshold effect was observed for body weight, with patients weighing > 80 kg exhibiting a 2.71-fold increase in thromboembolism risk (95% CI: 1.70–4.30). However, 58.96% of thromboembolism cases lacked weight data, which is a limitation that should be addressed in future studies.

Previous studies^16,27^ have shown that tamoxifen may increase the risk of venous thrombosis but has no significant effect or even a protective effect on arterial thrombosis (including coronary events), as it reduces coronary plaque in vivo, improves lipid levels, reduces C-reactive protein, and modulates nitric oxide production. In breast cancer treatment, the risk of major adverse cardiovascular events (MACE, including coronary artery disease, myocardial infarction, etc.) was significantly reduced in TAM users compared with aromatase inhibitors (AIs)^20^suggesting a potential cardiovascular benefit. However, we identified 28 thromboembolic signals by disproportionality analysis, with 11% being arterial thrombotic events (including coronary embolism), although dominated by pulmonary embolism (ROR = 5.59) and deep vein thrombosis (ROR = 5.17). The following discrepancy between this finding and previous studies may be due to the fact that most of the literature attributes arterial thromboembolism to traditional cardiovascular risk factors (e.g., myocardial infarction, arrhythmia) and does not comprehensively and systematically analyze the contribution of TAM to all types of arterial embolism. Moreover, when analyzed by gender stratification, men had a higher incidence of arterial thromboembolic events than women, and the mortality rate of arterial events was extremely high - coronary artery embolism had a 100% mortality rate (3/3). This is in contrast to the findings of previous studies, which tended to discuss overall thrombotic risk and did not discuss it by age groups, and further analysis of the factors influencing the cardiovascular events of tamoxifen is needed to clarify specific risk indicators.

Although thromboembolic risks associated with tamoxifen are already highlighted in its prescribing information, including a boxed warning, our study provides several important refinements to the existing safety knowledge. First, by employing a structured classification and prioritization framework (e.g., IME and DME categories), we offer a more nuanced understanding of thromboembolic signal strength, aiding in clinical triage. Second, the use of FAERS pharmacovigilance data captures long-term, real-world exposure patterns and patient subgroups not typically represented in controlled trials. Third, we detected several thromboembolic signals that are currently absent from drug labeling, indicating potential emerging risks. Together, these enhancements help bridge the gap between regulatory awareness and clinical risk mitigation.

In this study, disproportionality signals were further analyzed using a scoring scale to prioritize safety concerns and minimize unwarranted alerts. Clinical prioritization analysis of TAM-associated thromboembolic adverse events (AEs) revealed that most events were classified as important medical events (IMEs), highlighting their potential impact on patient health. Among all AEs, 57.1% were assigned intermediate priority, with pulmonary embolism (*n* = 123, score = 6), deep vein thrombosis (*n* = 79, score = 6), thrombosis (*n* = 62, score = 6), coronary artery embolism (*n* = 3, score = 6), and cerebral vein thrombosis (*n* = 24, score = 6) comprising the top five. Enhanced clinical monitoring is therefore recommended. Notably, three of the 28 AEs were identified as novel and unexpected safety signals not included in the current drug labeling. These signals comprised monoplegia (ROR = 7.94), retinal artery occlusion (ROR = 10.80), and atrial thrombosis (ROR = 6.12). This underscores the need for detailed analysis of existing patient records and strengthened post-marketing surveillance to enable real-time monitoring. The specific impact of TAM on these AEs and their underlying mechanisms remains insufficiently investigated and warrant further clinical exploration. Given the pervasive risk of TAM-associated thromboembolism, particularly with newly documented signals, clinicians should recognize these concerns as critical safety alerts.

In light of these findings, it is important to translate signal detection into concrete clinical strategies to mitigate thromboembolic risk. Based on the stratified risk profiles identified in this study, we recommend that male patients prescribed tamoxifen, particularly those over 65 years of age or with body weight exceeding 80 kg, undergo enhanced surveillance. This may include baseline and periodic Doppler ultrasound examinations for lower extremity deep vein thrombosis, symptom-based monitoring for signs such as dyspnea or unilateral limb swelling, and early cardiology or hematology consultation in patients with pre-existing cardiovascular or prothrombotic conditions^28^. In the event of new-onset symptoms suggestive of thromboembolism—such as chest pain, shortness of breath, focal neurological deficits, or limb edema—clinicians should promptly initiate diagnostic evaluation and consider temporary discontinuation of tamoxifen until the cause is clarified^29^. Furthermore, in male breast cancer patients, individualized risk–benefit assessments may support the substitution of aromatase inhibitors plus gonadotropin-releasing hormone analogues when the thrombotic risk is deemed excessive. These recommendations underscore the need for sex-specific, risk-adapted monitoring protocols and highlight a broader gap in existing clinical guidelines for tamoxifen safety management.

This study has several limitations inherent to the nature of spontaneous reporting systems. First, the FAERS database is a passive surveillance system that is subject to underreporting bias, particularly for less severe or subclinical events. As a result, the observed signals may overrepresent severe thromboembolic outcomes such as pulmonary embolism and coronary artery embolism, while underestimating the incidence of milder events. Second, a substantial proportion (58.96%) of thromboembolic cases lacked complete body weight data, which may have introduced bias into the risk estimation for overweight or obese individuals. Although sensitivity analyses were attempted to account for this, the absence of this key covariate limits the precision of our multivariable modeling. Third, despite the application of rigorous deduplication algorithms and standardized disproportionality methods, the FAERS database lacks denominator data (i.e., total number of exposed patients), which precludes absolute risk estimation. Finally, causality cannot be definitively established from signal detection results, and the findings should be interpreted as hypothesis-generating rather than confirmatory.

## Conclusion

The present pharmacovigilance study confirms that tamoxifen significantly increases the risk of arteriovenous thrombosis and that there are stratified differences in risk by sex, age, and weight. The identification of male-specific vulnerability, age-indexed growth patterns of risk, and high-signal thrombotic subtypes (e.g., cerebral venous thrombosis, ROR = 58.10) reshapes surveillance priorities for the TAM-treated population. Although the inherent limitations of spontaneous reporting systems affect the precision of risk quantification, the results of this study call for improved clinical surveillance protocols, including gender-specific screening, geriatric dose optimization, and targeted imaging assessment of high-risk events.

## Supplementary Information

Below is the link to the electronic supplementary material.

Supplementary Material 1

## Data Availability

All data generated or analysed during this study are included in this published article (and its Supplementary Information files). The FAERS datasets analyzed during the current study are available on FDA websites (https://fis.fda.gov/extensions/FPD-QDE-FAERS/FPD-QDE-FAERS.html).
