# Supplementary material for: Real-world assessment of thromboembolic risk associated with tamoxifen
Source: Sci Rep. 2025 Jul 30;15:27820. doi: 10.1038/s41598-025-13585-0 (PMC12310949; doi:10.1038/s41598-025-13585-0)
Supplement: Supplementary file 1 — Supplementary Material 1 [file 41598_2025_13585_MOESM1_ESM.docx]

**Supplementary tables**

**Table S1** Two-by-two contingency (2×2) table for disproportionality analyses.

|  | **Target adverse event** | **All other adverse events** | **total** |
| --- | --- | --- | --- |
| **Target drug** | a | b | a + b |
| **All other drugs** | c | d | c + d |
| **total** | a + c | b + d | a + b + c + d |

**Table S2** Algorithms used for signal detection.

|  | **Equation** | **Optional signal** |
| --- | --- | --- |
| **ROR** | ROR = (a/b) / (c/d) | a≥3，ROR>2，95%CI |
| **PRR** | PRR = [a / (a + b)] / [c / (c + d)]  χ^2^ = [(ad-bc)^2^(a+b+c+d)] / [(a + b)(c + d)(a + c)(b + d)] | a≥3，PRR>2，χ^2^>4 |
| **BCPNN** | IC = log₂ [a(a + b + c + d) / [(a + c)(a + b)]]  95CI = e^ln(IC)±1.96(1/a + 1/b + 1/c + 1/d)^0.5^ | IC025 > 0 |
| **EBGM** | EBGM = a(a + b + c + d) / (a + c) / (a + b)  95CI = e^ln(EBGM)±1.96(1/a + 1/b + 1/c + 1/d)^0.5^ | EBGM05 > 2, a ≥ 0 |

**Table S3** A rating scale assessing clinical priority of disproportionality signals.

| **Assessment items** | **2 points** | **1 point** | **0 point** |
| --- | --- | --- | --- |
| Number of target events (a) | >50 | 10–50 | <10 |
| Lower limit of ROR | >5 | 2–5 | 1–2 |
| Mortality proportion (%) | >50 | 25–50 | <25 |
| IMEs or DMEs | DME | IME | None |
| Biological plausibility | ++ | + | - |

++: adverse events (AEs) are mainly from the FDA Prescribing Information, the summary of product characteristics of canakinumab posted by the MHRA, Phase 2/3 RCTs, or systematic reviews, with biological plausibility. +: AEs are mainly from other clinical trials, observational studies, or case reports/series with potential biological plausibility. -: AEs only emerging from disproportionality analyses.

**Table S4** The distribution of positive signals of TAM-related thromboembolism based on preferred terms categorized by SMQ.

| **SMQ** | **report number** | **percent(%)** | **PTs number** | **percent(%)** |
| --- | --- | --- | --- | --- |
| Arterial Thromboembolism | 48 | 11.0 | 5 | 17.9 |
| Venous Thromboembolism | 310 | 70.9 | 19 | 67.9 |
| Thromboembolism of unspecified vascular type | 79 | 18.1 | 4 | 14.3 |

**Table S5** The distribution of positive signals of TAM-related thromboembolism based on preferred terms categorized by system organ classes.

| **SOC** | **report number** | **percent(%)** | **PTs number** | **percent(%)** |
| --- | --- | --- | --- | --- |
| Vascular disorders | 190 | 43.5 | 11 | 39.3 |
| Respiratory, thoracic and mediastinal disorders | 133 | 30.4 | 2 | 7.1 |
| Nervous system disorders | 63 | 14.4 | 5 | 17.9 |
| Eye disorders | 21 | 4.8 | 4 | 14.3 |
| Hepatobiliary disorders | 14 | 3.2 | 2 | 7.1 |
| Cardiac disorders | 6 | 1.4 | 2 | 7.1 |
| Blood and lymphatic system disorders | 6 | 1.4 | 1 | 3.6 |
| Gastrointestinal disorders | 4 | 0.9 | 1 | 3.6 |

**Table S6** Clinical priority assessing results of disproportionality signals.

| **PT** | ***n*** | **ROR_025_** | **Death (*n*)** | **IMEs/DMEs** | **Relevant evidence evaluation** | **Priority level (score)** |
| --- | --- | --- | --- | --- | --- | --- |
| Pulmonary Embolism | 123 | 4.68 | 10 | IME | ++ | moderate (6) |
| Deep Vein Thrombosis | 79 | 4.14 | 8 | IME | ++ | moderate (6) |
| Thrombosis | 62 | 2.61 | 3 | IME | ++ | moderate (6) |
| Cerebral Venous Thrombosis | 24 | 38.82 | 0 | IME | ++ | moderate (6) |
| Ischaemic Stroke | 20 | 3.15 | 3 | IME | ++ | moderate (5) |
| Peripheral Artery Thrombosis | 12 | 20.88 | 0 | NA | ++ | moderate (5) |
| Pulmonary Thrombosis | 10 | 2.18 | 0 | IME | ++ | moderate (5) |
| Portal Vein Thrombosis | 9 | 6.52 | 1 | IME | ++ | moderate (5) |
| Monoplegia | 8 | 3.97 | 0 | IME | + | Weak (3) |
| Superior Sagittal Sinus Thrombosis | 7 | 17.53 | 0 | IME | ++ | moderate (5) |
| Blindness Transient | 7 | 2.00 | 0 | IME | + | Weak (3) |
| Embolism Venous | 6 | 3.99 | 0 | IME | ++ | Weak (4) |
| Antiphospholipid Syndrome | 6 | 7.59 | 0 | IME | ++ | moderate (5) |
| Thrombophlebitis | 6 | 3.24 | 0 | NA | ++ | Weak (3) |
| Retinal Artery Occlusion | 6 | 4.85 | 0 | IME | ++ | Weak (4) |
| Jugular Vein Thrombosis | 6 | 6.72 | 0 | NA | ++ | Weak (4) |
| Budd-Chiari Syndrome | 5 | 36.47 | 0 | IME | ++ | moderate (5) |
| Retinal Vein Occlusion | 5 | 3.30 | 0 | IME | ++ | Weak (4) |
| Venous Thrombosis Limb | 5 | 4.00 | 0 | NA | ++ | Weak (3) |
| Mesenteric Vein Thrombosis | 4 | 6.61 | 0 | IME | ++ | moderate (5) |
| Subclavian Vein Thrombosis | 4 | 5.59 | 0 | IME | ++ | moderate (5) |
| Transverse Sinus Thrombosis | 4 | 12.05 | 0 | IME | ++ | moderate (5) |
| Superficial Vein Thrombosis | 4 | 11.15 | 0 | NA | + | Weak (3) |
| Coronary Artery Embolism | 3 | 14.12 | 3 | IME | + | moderate (6) |
| Ophthalmic Vein Thrombosis | 3 | 17.86 | 0 | IME | ++ | moderate (5) |
| Vena Cava Thrombosis | 3 | 2.51 | 0 | IME | + | Weak (3) |
| Atrial Thrombosis | 3 | 1.97 | 0 | IME | ++ | Weak (3) |
| Axillary Vein Thrombosis | 3 | 11.56 | 0 | NA | + | Weak (3) |
